# Impact of Serum/Xeno‐Free Medium and Cytokine Supplementation on CAR‐T Cell Therapy Manufacturing in Stirred Tank Bioreactors

**DOI:** 10.1002/biot.70114

**Published:** 2025-09-09

**Authors:** Pedro Silva Couto, Dale J. Stibbs, Pierre Springuel, Ursula Schultz, Manuel Effenberger, Stephen Goldrick, Sergio Navarro‐Velázquez, Manel Juan, Laura Herbst, Bastian Nießing, Katrin Mestermann, Carmen Sanges, Michael Hudecek, Qasim A. Rafiq

**Affiliations:** ^1^ Department of Biochemical Engineering University College London London UK; ^2^ Sartorius CellGenix GmbH Freiburg im Breisgau Germany; ^3^ Immunology Department – Immunotherapy Section Hospital Clínic Barcelona Barcelona Spain; ^4^ Fundació De Recerca Clínic Barcelona‐Institut D'investigacions Biomèdiques August Pi i Sunyer (FCRB‐IDIBAPS) Barcelona Spain; ^5^ HSJD‐Clínic Immunotherapy Platform Barcelona Spain; ^6^ Fraunhofer Institute for Production Technology IPT Aachen Germany; ^7^ Medizinische Klinik und Poliklinik II Lehrstuhl Für Zelluläre Immuntherapie Universitätsklinikum Würzburg Würzburg Germany; ^8^ Fraunhofer‐Institut Für Zelltherapie Und Immunologie Außenstelle Zelluläre Immuntherapie Würzburg Germany

**Keywords:** ATMP, biomanufacturing, CAR‐T, GMP, serum free, stirred‐tank bioreactor, xeno‐free

## Abstract

Chimeric antigen receptor T‐cell (CAR‐T) therapies have demonstrated clinical efficacy in treating haematological malignancies, resulting in multiple regulatory approvals. However, there is a need for robust manufacturing platforms and the use of GMP‐aligned reagents to meet the clinical and commercial demands. This study investigates the impact of serum/xeno‐free medium (SXFM) and cytokine supplementation on CAR‐T cell production in static and agitated culture systems, using 24‐well plate G‐Rex vessels and 500 mL stirred tank bioreactors (STRs), respectively. Under static conditions, SXFM media supported CAR‐T cell expansion with growth kinetics comparable to foetal bovine serum, FBS‐based RPMI, irrespective of the cytokine supplementation (IL‐2 or the combination of IL‐7 and IL‐15). In contrast, when the expansion was conducted using STRs, several differences were observed with SXFM. Particularly, when supplemented with IL‐2 SXFM, it increased transduction efficiency, supporting accelerated proliferation relative to FBS‐containing RPMI. Additionally, SXFM maintained a higher CD4:CD8 ratio at harvest, a feature associated with improved clinical outcomes. No significant differences were observed in the CAR‐T cell populations' differentiation status or activation and exhaustion profiles across the conditions. These results suggest that SXFM enables CAR‐T cell manufacturing in STRs, improving key quality attributes such as transduction efficiency, growth kinetics, and CD4:CD8 ratio compared to FBS‐supplemented medium.

## Graphical Abstract and Lay Summary

Medium formulation and cytokine combinations strongly influence CAR‐T cell growth, metabolism, phenotype, and cytotoxicity in static and agitated platforms, defining critical parameters for scalable manufacturing using stirred tank bioreactors.

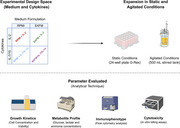

**Abbreviations**
ALL: acute lymphoblastic leukaemia
ATMP: advanced therapy medicinal product
BCMA: B‐cell maturation antigen
CAR‐T: chimeric antigen receptor T‐cell
CD: cluster of differentiation
CQAs: critical quality attributes
CR: complete response
CRS: cytokine release syndrome
DO: dissolved oxygen
EMA: European Medicines Agency
FBS: foetal bovine serum
FDA: Food and Drug Administration
GMP: good manufacturing practice
hPL: human platelet lysate
ICANs: immune effector cell‐associated neurotoxicity syndrome
IL: interleukin
LAG‐3: lymphocyte activation gene‐3
LBCL: large B‐cell lymphoma
MCL: mantle cell lymphoma
MM: multiple myeloma
NHL: non‐Hodgkin's lymphoma
NMPA: National Medical Products Administration
ORR: overall response rate
PBMC: peripheral blood mononuclear cells
PEI: polyethyleneimine
Po: power number
QC: quality control
RPMI: Roswell Park Memorial Institute Medium
SPSS: statistical package for the social sciences
STR: stirred tank bioreactor
TCR: T‐cell receptor
TRUCKs: T cells redirected for antigen‐unrestricted cytokine‐initiated killing

## 1 Introduction

*Ex vivo* gene therapies, such as chimeric antigen receptor (CAR)‐T cell therapies, have revolutionised the treatment landscape for haematological malignancies, providing transformative options for patients who have exhausted all other therapeutic avenues [1]. As shown in Table 1, there are currently seven US Food and Drug Administration (FDA) approved autologous CAR‐T cell therapies, with several additional products approved by other regulatory agencies globally. For adult patients, 10 to 500 × 10^6^ CAR‐positive (CAR+) cells are required to meet the dosage specifications Table 1 [2, 3, 4, 5, 6, 7, 8, 9, 10, 11].

**TABLE 1:** Overview of globally approved CAR‐T cell therapies, highlighting key regulatory milestones, target antigens, therapeutic indications, companies responsible for their commercialisation alongside product efficacy and safety information.

Product name	Year of approval	Target receptor	Dose size (10^6^ cells)	Company	Regulatory body	Approved indications	Efficacy	Safety	Ref.
Kymriah	2017	CD19	10–250	Novartis	FDA, EMA, MHRA, MHLW, MFDS	B‐cell ALL, DLBCL	ORR: 82.5% CR: 63% Relapse: 11/52	CRS:77% ICANs: 40%	[2]
Yescarta	2017	CD19	200	Kite Pharma (Gilead)	FDA, EMA, MHRA, NMPA	NHL, LBCL, FL	ORR: 83% CR: 58% Relapse: 13/65	CRS:85% ICANs: 33%	[3]
Tecartus	2020	CD19	100–200	Kite Pharma (Gilead)	FDA, EMA, MHRA	MCL	ORR: 87% CR: 62% Relapse: 9/43	CRS:94% ICANs: 35%	[4]
Breyanzi	2021	CD19	50–110	BMS (Juno Therapeutics)	FDA, EMA, MHRA, MHLW	LBCL	ORR: 73% CR: 53% Relapse: 18/79	CRS:63% ICANs: 30%	[5]
Abecma	2021	BCMA	300–510	BMS (Celgene)	FDA, EMA, MHRA	MM	ORR: 72% CR: 28% Relapse: 21/65	CRS:89% ICANs: 40%	[6]
Carvykti	2022	BCMA	100	Janssen Biotech, Inc.	FDA, EMA, MHRA	MM	ORR: 97.9% CR: 82.5% Relapse: 5/97	CRS:95% ICANs: 25%	[7]
Carteyva	2021	CD19	Unknown	JW Therapeutics	NMPA	LBCL, FL, MCL	NA	NA	[8]
Fucaso	2023	BCMA	Unknown	Innovent Biologics & IASO Biotech	NMPA	MM	ORR: 95% CR: 76.9% Relapse: NA	CRS:93.2% ICANs: 1.9%	[9, 10]
NexCAR19	2023	CD19	Unknown	ImmunoACT	CDSCO	B‐cell ALL, LBCL	NA	NA	[12]
Aucatzyl	2024	CD19	410	Autologous	FDA	B‐cell ALL	ORR: 76% CR: 45% Relapse: 14/60	CRS:68% ICANs: 29%	[13]
Qartemi	2025	CD19	Unknown	Immuneel Therapeutics	CDSCO	B‐NHL	NA	NA	—

**Regulatory bodies**: FDA (Food and Drug Administration, USA), EMA (European Medicines Agency, European Union), MHRA (Medicines and Healthcare products Regulatory Agency, UK), MHLW (Ministry of Health, Labour and Welfare, Japan), NMPA (National Medical Products Administration, China), CDSCO (Central Drugs Standard Control Organisation, India). **Indications**: B‐cell ALL (B‐cell Acute Lymphoblastic Leukemia), DLBCL (Diffuse Large B‐Cell Lymphoma), NHL (Non‐Hodgkin Lymphoma), LBCL (Large B‐Cell Lymphoma), FL (Follicular Lymphoma), MCL (Mantle Cell Lymphoma), MM (Multiple Myeloma), B‐NHL (B‐Cell Non‐Hodgkin Lymphoma). NA, not available.

The growing demand for consistent, large‐scale CAR‐T cell manufacturing has highlighted the challenges associated with raw materials, particularly culture supplements such as foetal bovine serum (FBS). Previous studies have shown that FBS can compromise manufacturing consistency compared to alternatives such as serum/xeno‐free media (SXFM) or media supplemented with human platelet lysate (hPL) [14, 15].

One major concern of FBS is the risk of transmitting zoonotic pathogens, such as viruses and prions, which can lead to infections in patients receiving cell and gene therapy‐based therapies. Additionally, FBS can introduce immunogenic agents, potentially triggering immune reactions that compromise therapeutic efficacy. FBS's undefined and variable composition contributes to batch‐to‐batch inconsistencies, affecting reproducibility and quality control in cell cultures [16]. It poses a critical challenge due to its regulatory burden, as agencies like the FDA and European Medicines Agency (EMA) impose stringent documentation and testing requirements to ensure the absence of contaminants and pathogens from this animal‐derived product. These additional safety measures increase the manufacturing process's cost and complexity and prolong timelines for good manufacturing practices (GMP) compliance. Alternatives such as hPL or human serum supplementation reduce some risks but may not eliminate the challenge of batch‐to‐batch variability [17, 18]. In addition, the limited and unstable supply of FBS creates supply chain vulnerabilities and raises sustainability concerns for large‐scale cell and gene therapy manufacturing [18].

In addition to medium formulation, cytokine supplementation is of critical importance during the CAR‐T manufacturing processes due to their impact on T‐cell biology and overall final product quality [19, 20, 21, 22, 23, 24]. Although IL‐2 has historically been linked to enhanced T‐cell growth kinetics, recent studies have challenged this assumption, with some failing to confirm its enhanced performance in promoting T‐cell expansion over other cytokines [25, 26]. In addition, broader literature indicates that IL‐2 is associated with skewing T cells toward an exhausted phenotype [27], preferentially supporting CD8+ over CD4+ T cell expansion [19], and promoting a more differentiated phenotype [24], compared to IL‐7/IL‐15.

In contrast, IL‐7 enhances the expansion of naïve and central memory T cells, promoting a less differentiated phenotype that is associated with improved engraftment and persistence in vivo [20]. IL‐15 plays a crucial role in supporting the homeostatic proliferation of memory T cells while maintaining stem‐like properties that correlate with enhanced antitumor activity and durability [28]. Literature suggests that, when combined, IL‐7 and IL‐15 promote a less terminally differentiated, memory‐enriched CAR‐T cell product, which has been linked to superior in vivo expansion and persistence compared to IL‐2‐driven cultures [20, 29].

Consequently, cytokine selection during CAR‐T manufacturing directly impacts phenotypic composition, persistence, and therapeutic efficacy, making it a critical parameter in optimising manufacturing protocols for improved clinical outcomes. Given that most studies evaluating the impact of cytokines are performed in the presence of FBS and static conditions, there is a current knowledge gap in understanding how these may impact the final product quality when the expansion occurs under SXF conditions.

Multiple systems used for clinical manufacturing of CAR‐T, such as T‐flasks, bags or G‐Rex, are often described as open systems, requiring a high degree of manual manipulation and necessitating high‐grade clean rooms and have an inherently high risk of contamination. Widely used in traditional biotechnology to manufacture commercially successful therapies (including recombinant proteins, antibody‐drug conjugates [30], and viral vectors [31, 32]), stirred tank reactors (STRs) offer robust control over critical biophysical parameters such as temperature, dissolved oxygen (DO), pH and agitation speed. Precise control capabilities over these parameters minimise batch‐to‐batch variability. In addition, STRs support a range of feeding strategies, including batch, fed‐batch and perfusion [33, 34, 35, 36]. These features enable precise control of process parameters, ensuring consistent critical quality attributes (CQAs) of the product, which are essential for complying with GMP guidelines and regulatory requirements. Moreover, STRs are compatible with closed‐system configurations, which reduce contamination risks and lower the cleanroom classification requirements (e.g., from Grade B to Grade C environments) and can be scaled‐up to several thousand litres [37, 38, 39]. Importantly, these have the potential to enable end‐to‐end manufacturing, where the entire process is conducted in the reactors’ vessel or a modular approach by connecting with other technologies developed to conduct gene delivery [40, 41] or harvesting [42].

Given the influence of medium formulation, cytokine supplementation, and expansion platform on CAR‐T cell characteristics, this study investigates the use of SXFM supplemented with either IL‐2 or a combination of IL‐7 and IL‐15 to support CAR‐T cell manufacturing, both in static and agitated conditions.

## 2 Materials and Methods

### 2.1 T‐Cell Isolation

Following the manufacturer's protocol, T‐cells were isolated from peripheral blood mononuclear cells (PBMCs) using pan T‐cell isolation kits (Miltenyi Biotec, Germany). Briefly, upon arrival, the cell contents of a leukopak (BioIVT, UK) were diluted 1:1 using MACS buffer consisting of a MACS bovine serum albumin stock solution (Miltenyi Biotec, Germany 5% (v/v) and autoMACS rinsing solution (Miltenyi Biotech, Germany) 95% (v/v). An initial centrifugation was performed to pellet the PBMCs, and they were resuspended in the buffer mentioned previously at a concentration of 2.5 × 10^8^ cells.mL^−1^. The cell preparation was incubated with a T‐cell biotin‐antibody cocktail (Miltenyi Biotec, Germany) followed by a pan T‐cell microbead cocktail (Miltenyi Biotec, Germany) both for 5 min at 4°C. The resulting suspension was loaded into pre‐rinsed LS columns (Miltenyi Biotec, Germany), and the negative fraction containing the cells of interest was collected. These were then centrifuged (400 g, 5 min) and resuspended in CS10 (Biolife Solutions, USA) at a concentration of 50 x 10^6^ cells.mL^−1^ of cryoprotectant. The cells were transferred to a −80°C freezer overnight in CoolCell (Corning, USA) and moved to liquid nitrogen tanks for long‐term storage within 24 h. A total of three biological donors were isolated and used throughout this work.

### 2.2 Medium Formulations

T‐cells were expanded in two mediums: one, composed of Roswell Park Memorial Institute (RPMI) medium supplemented (Gibco, UK) with 10% (v/v) FBS (Gibco, UK) and 2 mM of L‐glutamine (Gibco, UK). In addition, one serum xeno‐free medium formulation called CellGenix Advanced TCM (SXFM from now onward) was evaluated (Sartorius, Germany). For T‐cell activation and growth, cytokines were added to the medium as either IL‐2 (Miltenyi Biotec, Germany) or a combination of IL‐7/IL‐15 (Sartorius, Germany) at a concentration of 30 IU.mL^−1^ and 10 ng.mL^−1^ of culture medium, respectively. Unless stated otherwise, cytokine usage was maintained at the concentrations referred to in this section.

### 2.3 Thawing and Activation

Cell thawing was completed using medium pre‐warmed in a 37°C water bath. Upon thawing, the cryoprotectant agent was washed using a centrifugation cycle (400 g, 5 min) followed by a cell resuspension step to keep the cells between the 1–2 × 10^6^ cells.mL^−1^ target density. Activation was performed 24 h post‐thawing using TransAct (Miltenyi Biotecm, Germany) in the presence of either IL‐2 (Miltenyi Biotec, Germany) or a combination of IL‐7/IL‐15 (Sartorius, Germany) as described in **Section** **2.2**.

### 2.4 Transduction

A third‐generation lentiviral vector system was used to perform CAR transgene knock‐in using manufacturing methods described previously [43]. Briefly, vector was prepared starting with a 5‐day expansion of HEK 293T cells seeded at a density of 5,000 cells.cm^−2^. For the transfection, the DNA plasmid ratio transfer vector:Gag‐Pol:REV:VSVG was 4:2:1:1.2, with the transfer vector encoding a CAR gene using a DNA:PEI ratio of 1:2.75. DNA and PEI (Sartorius, Germany) were mixed and incubated for 15 min at room temperature to allow for complexation. After that, the solution was added dropwise on top of the cells. A full medium exchange was performed 6 h post‐transfection to minimise cytotoxicity of the PEI. The supernatant was collected 48 h post‐transfection, then filtered through a 0.45 µM filter (Merck, Germany) and concentrated using Lenti‐X (TakaraBio, Japan).

A spinoculation‐based process was conducted using retronectin‐coated 6‐well plates (4 µg.cm^−2^) (TakaraBio, Japan) using a centrifugation process (1,000 *g*, 40 min at 33°C) using the respective medium as highlighted above. The transduction step was performed using a cell suspension of 2 × 10^6^ cells.mL^−1^ using a total volume of 2 mL per well. The plates were incubated for 24 h and subsequently seeded in cell culture flasks at 0.5 × 10^6^ cells. mL^−1^ incubated at 37°C and 5% CO for 3 days (seed train) and then transferred to the expansion platforms as indicated in Section 2.5.

### 2.5 Cell Expansion

#### 2.5.1 Static Studies

**FIGURE 1:**
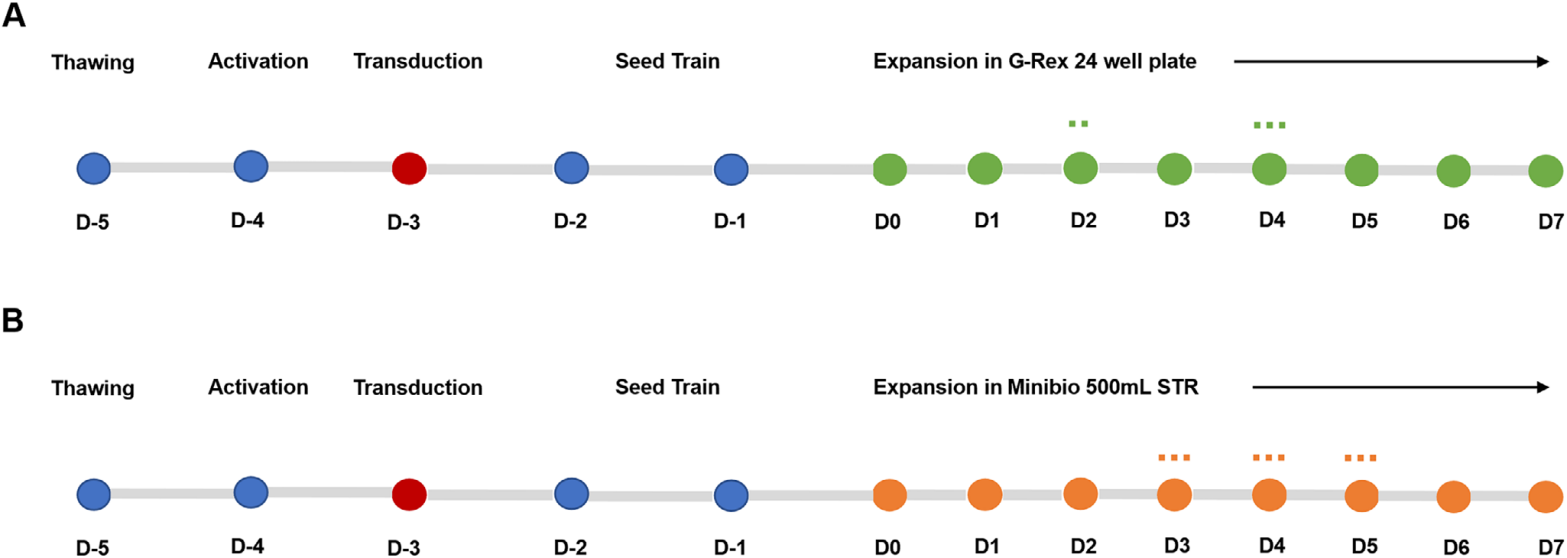
Process diagram for the CAR‐T manufacturing processes used in this work. D‐5 to D‐1 was conducted in T‐flask, except for D‐3, where the spinoculation was performed in retronectin‐coated 6‐well plates. The expansion cycle was conducted either in the (A) static G‐Rex 24 well plate platform or using the (B) STR Minibio 500 mL. Cytokine supplementation was performed on Day 2 in the static G‐Rex 24 well plate as represented by the two dots. Medium exchanges/additions were performed on Day 4 in the static G‐Rex 24 well plate and on Days 3, 4, and 5 in the STR platform, as represented by the three dots.

To evaluate CAR‐T cell expansion in static conditions, G‐Rex 24 well plates (Wilson Wolf, USA) were used following previously described protocols [44]. Briefly, CAR‐T cells were seeded at 0.5 × 10^6^ cells.cm^−2^, with the volume of each well topped up to 8 mL. Cytokines were added 2 days after seeding, and a 75% medium exchange containing cytokines was performed 4 days into the expansion phase (Figure 1A). The static culture studies used one biological donor and three technical replicates.

#### 2.5.2 Stirred Tank Bioreactor Studies

Previous work has demonstrated the feasibility of STR platforms such as the ambr250 (Sartorius, Germany) for CAR‐T expansion [37, 38, 45, 46]. In this work, a MiniBio 500 mL platform (Getinge, Netherlands) was used to evaluate the feasibility of using STRs to manufacture CAR‐T therapies. The bioreactor vessels were equipped with a 71 mm diameter vessel with a 3‐blade marine impeller (*D* = 28 mm) with a power number (Po) of 1.5. The minimum and maximum working volumes were 100 and 400 mL, respectively.

The specific power per unit volume (P/V) was calculated using Equation (1) and used as scale‐up criteria. Agitation speed was adjusted to maintain a P/V value of 74 × 10^−4^ W.kg^−1^, consistent with conditions previously optimised in ambr250 studies [37, 38, 45, 46]. Based on this target, the nominal agitation speed in the 500 mL STR was set to 250 rpm (Table 2) to support scale‐up while preserving comparable mixing conditions.

**TABLE 2:** Summary of the bioreactor parameters used to set the agitation speed in the 500 mL stirred tank bioreactor used in this work.

Parameter	Ambr250 Unbaffled [38]	MiniBio 500mL
P_o_ (dimensionless)	2.07	1.5
Ρ (kg.m^−3^)	998	998
Impeller diameter (m)	3.0 × 10^−2^	0.28 × 10^−2^
Volume (m^3^)	250	250
P (W)	0.00186	0.00186
Agitation speed (rpm)	200	250

During the bioreactor‐based culture, pH, DO, temperature, and the headspace gas flow were measured continuously and controlled throughout the experiment. Although pH was controlled at 7.2 ± 0.2 using CO_2_ with a flow rate with a maximum nominal value of 100 mL.min^−1^ and a 1 M solution of NaHCO_3_ (Sigma–Aldrich, Germany). The 50% DO set point was maintained using headspace gassing with air and oxygen. The experiment continuously measured and monitored pH, DO, temperature and headspace gas flow. The headspace air and O_2_ were regulated in the function of DO control with flow rates up to a maximum of 100 mL.min^−1^. In this cascade, O_2_ was supplemented to the culture only when air was insufficient to keep the DO value above the set point.

Following the initial 5‐day period consisting of thawing, transduction and pre‐expansion, CAR‐T cells were seeded into 100 mL of culture medium at a concentration of 0.5 × 10^6^ cells.mL^−1^. Three primary T‐cell donors were included to account for the biological variability expected in an autologous process. In this work, a fed‐batch process was performed using the following steps: (1) 100 mL of medium addition on Day 3, (2) 50 mL medium addition on Day 4, followed by (3) a 40% medium exchange on Day 5. In summary, the manufacturing methods used are represented in Figure 1B.

### 2.6 Analytical Techniques

#### 2.6.1 Cell Counts

Cell concentration and viability were assessed using a NucleoCounter NC‐3000 (Chemometec, Denmark) and the NucleoView software, which employs imaging analysis for these measurements. Cell counting and viability determination was conducted with Via1‐Cassettes (Chemometec, Denmark), which contain acridine orange (AO) and 4′,6‐diamidino‐2‐phenylindole (DAPI). AO, a membrane‐permeable dye, binds to cell nuclei, while DAPI selectively stains non‐viable cells with compromised membranes. Every time sampling was performed, 200 µL of the cell suspension was transferred into a reaction tube, followed by mixing using a vortex.

#### 2.6.2 Metabolite Analysis

Samples were taken daily from the bioreactors and G‐Rex 24 well plates to determine the metabolite concentration. To eliminate cells and debris, samples underwent centrifugation at 350 g for 5 min before being stored at −20°C. Before analysis, the frozen samples were thawed in a 37°C water bath and processed using the CuBiAn Bioanalyzer (Optocell, Germany), following the manufacturer's operating guidelines.

#### 2.6.3 Immunophenotype

To assess the nature of the T‐cell populations at the beginning of the expansion and at harvest, a flow cytometric analysis was performed on fresh cell samples at collection using a BD LSRFortessa X‐20 flow cytometer (BD Biosciences, UK). The list of conjugated antibodies used CD3‐BUV395 (BD Biosciences, UK), CD4‐BUV805 (BD Biosciences, UK), CD8‐APC‐Cy7 (BD Biosciences, UK), CCR7‐BV421 (BD Biosciences, UK), CD45RO‐PE‐Cy7 (BD Biosciences, UK), CD56‐BV605 (Biolegend, UK), CD34‐AlexaFluor647 (R&D Systems, USA) encoded in the anti‐CD19 CAR construct, CD69‐FITC (Biolegend, UK), PD‐1‐PE (Biolegend, UK), LAG‐3‐BV711 (Biolegend, UK) and Live/Dead‐UV511 (Invitrogen, UK) was set to capture information in regards to T‐cell subsets, differentiation, activation and exhaustion. A minimum of 100,000 events were acquired for each condition to ensure that the CAR+ fraction contained at least 10,000 events. The gating strategy featured fluorescence minus one controls for CCR7, CD45RO, CD56, CAR, CD69, PD‐1 and LAG‐3.

#### 2.6.4 In Vitro Cytotoxicity

In preparation for this assay, CAR+ T cells were isolated on the day of harvesting using the CD34 magnetic isolation kit following the manufacturer's instructions (Miltenyi Biotec, Germany). Briefly, cell suspensions were incubated with FcR Blocking Reagent and CD34 MicroBeads (Miltenyi Biotec, Germany), followed by washing and filtration through a MACS LS column (Miltenyi Biotec, Germany). The column was washed to remove unbound cells, and CD34+ cells were eluted upon removal from the magnetic field. The enriched CD34+ population was subsequently used for downstream CAR‐T assays.

Following the manufacturer's guidelines, an in vitro killing assay was conducted using the Incucyte S3 live‐cell analysis system (Sartorius, Germany). Briefly, a 1:1 ratio of target to effector cells was maintained in a 2‐day co‐culture, using the Incucyte Nuclight green‐labelled CD19‐positive NALM6 target cells. Given its representativeness of B cells, this cell type is commonly used in cytotoxicity assays against anti‐CD19 CAR constructs. Before the assay, NALM6 were thawed and expanded with seeding density set at 0.5 × 10^6^ cells.mL^−1^ and passaged to keep a viable cell concentration below 2.0 × 10^6^ cells.mL^−1^. The medium formulations used in this study matched those used for CAR‐T cell manufacturing.

#### 2.6.5 Statistical Analysis

Statistical analysis for this study was conducted using SPSS (IBM, USA). The selection of statistical tests was based on the underlying hypothesis of each experiment. When data did not meet the assumptions for parametric testing, appropriate non‐parametric alternatives were applied. The comparisons herein assessed were evaluated using a repeated measures one‐way ANOVA. When significant effects were observed, Bonferroni's correction was applied for post hoc pairwise comparisons. Statistical significance was defined at *p* values < 0.05, with significance levels represented as *p < 0.05, **p < 0.01, ***p < 0.001 and ****p < 0.0001.

### 2.7 Equations

#### 2.7.1 Power per Unit Volume

(1) $$\frac{P}{V}=\frac{P_{0}.N^{3}.\;D^{5}}{V}$$

*P* (W) represents the power input, *Po* is the power number (dimensionless), *ρ* (kg.m^−3^) is the density of the medium (here assumed to have the same physical properties as water), *N* (rev.s^−1^) is the impeller speed, *D* (m) is the impeller diameter and *V* is the volume of medium (*L*) in the bioreactor at the end of the culture.

#### 2.7.2 Doubling Time

(2) $$t_{d}=\frac{Ln_{2}}{\mu }$$

The numerator represents the natural log of 2, and *μ* denotes the specific growth rate (*d* ^‒1^).

#### 2.7.3 Growth Rate

(3) $$\mu =\frac{Ln\left(\frac{cx\left(t\right)}{cx\left(0\right)}\right)}{\Delta t}$$

Here, *μ* denotes the specific growth rate (*d* ^‒1^), while *Cx*(*t*) and *Cx*(0) correspond to the total cell count at the conclusion and initiation of the exponential growth phase, respectively. The variable *t* (*d*) represents time.

#### 2.7.4 Fold Increase

(4) $$FI=\frac{cx\left(f\right)}{cx\left(0\right)}$$

*Cx*(*t*) and *Cx*(0) denote the total cell count at the end and start of the process, respectively.

#### 2.7.5 Metabolic Production/Consumption Rate

(5) $$q_{met}=\frac{\mu }{cx\left(0\right)}\times \frac{C_{met\left(t\right)}-C_{met\left(0\right)}}{e^{\mu t}-1}$$

In this context, q_met_ (pmol.cell^−1^.d^−1^) refers to the specific metabolic rate, while μ denotes the specific growth rate (*d* ^‒1^). C_met_(t) and C_met_(0) indicate the metabolite concentrations (mmol.L^−1^) at the end and start of the exponential growth phase, respectively. Cx(0) represents the total cell count at the beginning of the exponential phase, and t corresponds to time (*d*).

## 3 Results

### 3.1 CAR‐T Expansion in Static Conditions

Several static platforms are available for CAR‐T cell manufacturing, such as flasks, G‐Rex, plates, and culture bags. The G‐Rex platform was selected for this initial evaluation due to its ability to expand immune cells in a static environment, supporting growth from 1 × 10^6^ to 250 × 10⁶ cells across different scales [44, 47, 48]. Since the literature indicates that low oxygen concentrations can negatively affect T‐cell differentiation, the G‐Rex platform was selected for this study [49, 50, 51]. Its gas‐permeable membrane was designed to maintain higher DO levels in culture, which could give it a potential advantage over traditional static culture systems.

**FIGURE 2:**
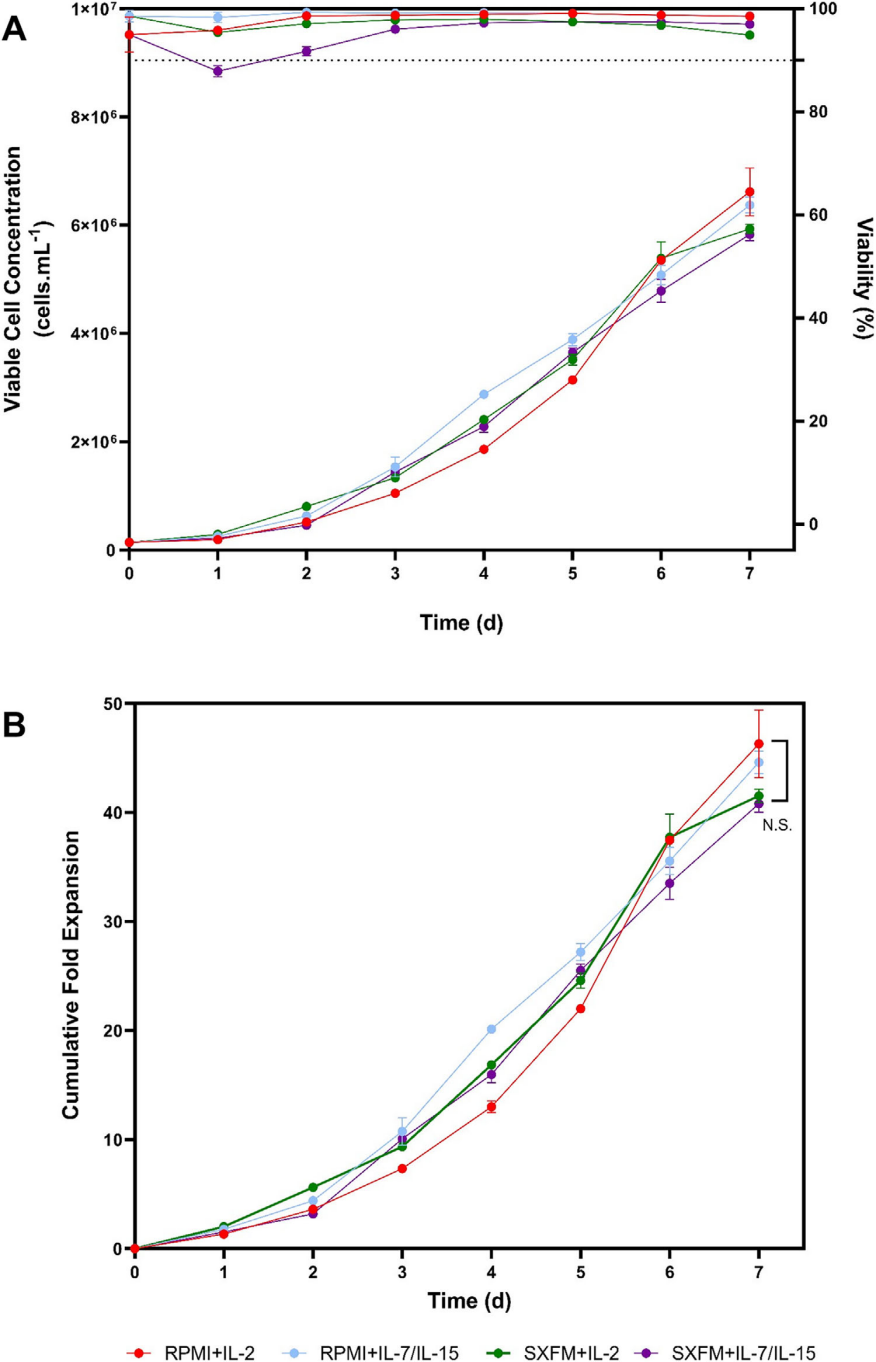
(A) Viable cell concentration and viability and (B) cumulative fold expansion during the static G‐Rex process. Points indicate the mean, with error bars representing one standard deviation (*N* = 3).

Figure 2A represents the growth profile of the cell populations across the experimental conditions. It was observed that irrespective of the medium and cytokine combination used, there were no differences with respect to cell yield generated at the harvesting point (*p* > 0.05). The maximum cell concentration ranged from 5.83 to 6.62 × 10^6^ cells.mL^−1^, and the total cell yield from 46.6 to 51.1 × 10^6^ cells. These processing conditions corresponded to cumulative fold increase levels between 39 and 49.6 (Figure 2B), while growth rates and doubling times reported within this design space were between 0.460 d^−1^ to 0.488 d^−1^ and 34.72 d to 35.88 h, respectively (Table S1).

**FIGURE 3:**
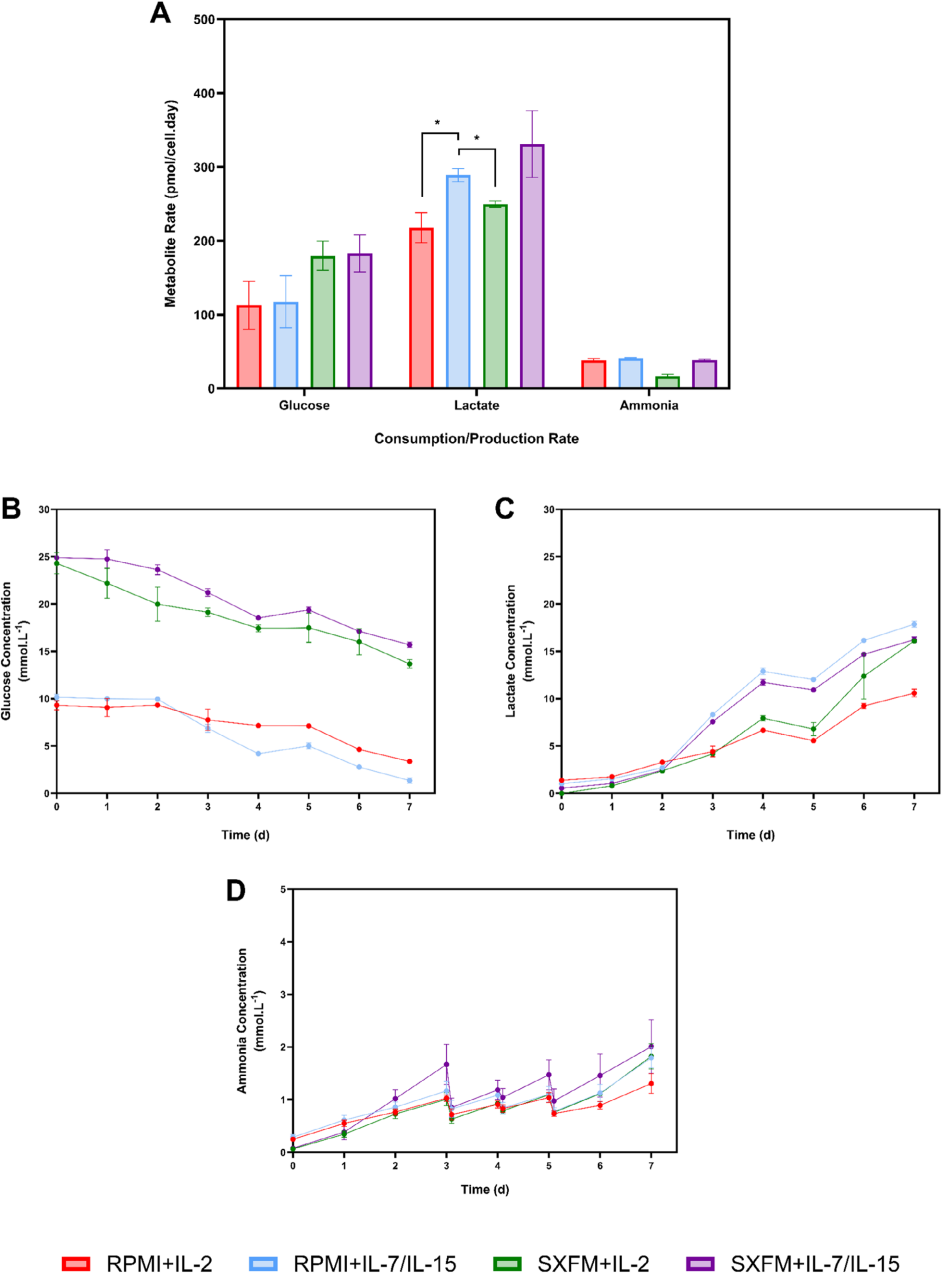
(A) Representation of the metabolite consumption and production rate and the concentration profiles for (B) glucose, (C) lactate and (D) ammonia in mmol.L^‒1^ during the static G‐Rex process. Vertical bars and points indicate the mean, with error bars representing one standard deviation (*N* = 3).

The comparable growth kinetics observed across experimental conditions were mirrored by similarities in the metabolic consumption and production profiles (Figure 3A). The only exception was with lactate, where IL‐2 led to a lower production rate compared to IL‐7/15. The metabolite analysis revealed that glucose levels approached depletion by the end of the expansion stage in conditions using RPMI, whereas it remained above 10 mmol.L^−1^ in SXFM (Figure 3B). Despite the similarities found in growth kinetics, lactate concentrations at the end of the expansion time ranged between 8 and 18 mmol.L^−1^, suggesting that different medium and cytokine combinations may influence the pathway cells use to metabolise glucose (Figure 3C). During the expansion window, ammonia levels peaked at 2 mM, suggesting that this metabolite was unlikely to contribute to growth inhibition (Figure 3D).

**FIGURE 4:**
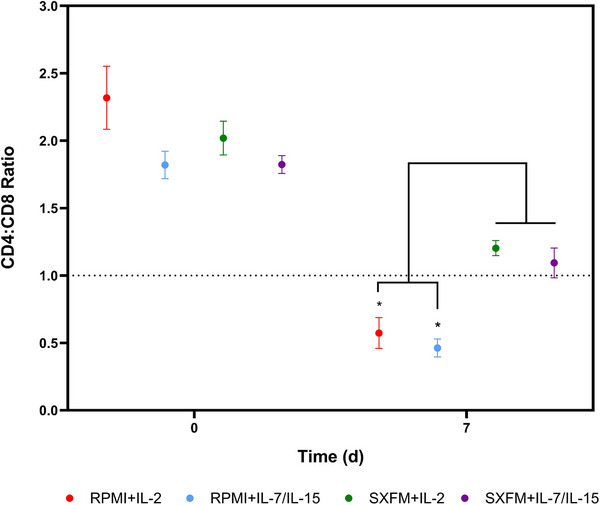
CD4:CD8 ratio in the T‐cell populations at seeding and harvesting for the static study. Data represented with the mean, with error bars representing one standard deviation (*N* = 3).

Regarding the phenotype observed across the experimental conditions, Figure 4 demonstrates that SXFM resulted in more balanced CD4:CD8 ratios than the RPMI groups despite the similarities observed at the start of the expansion phase (D0). Across the board, there was a decrease in the CD4:CD8 ratios throughout the expansion phase, indicating that CD8 cells grow faster than CD4 [52].

**FIGURE 5:**
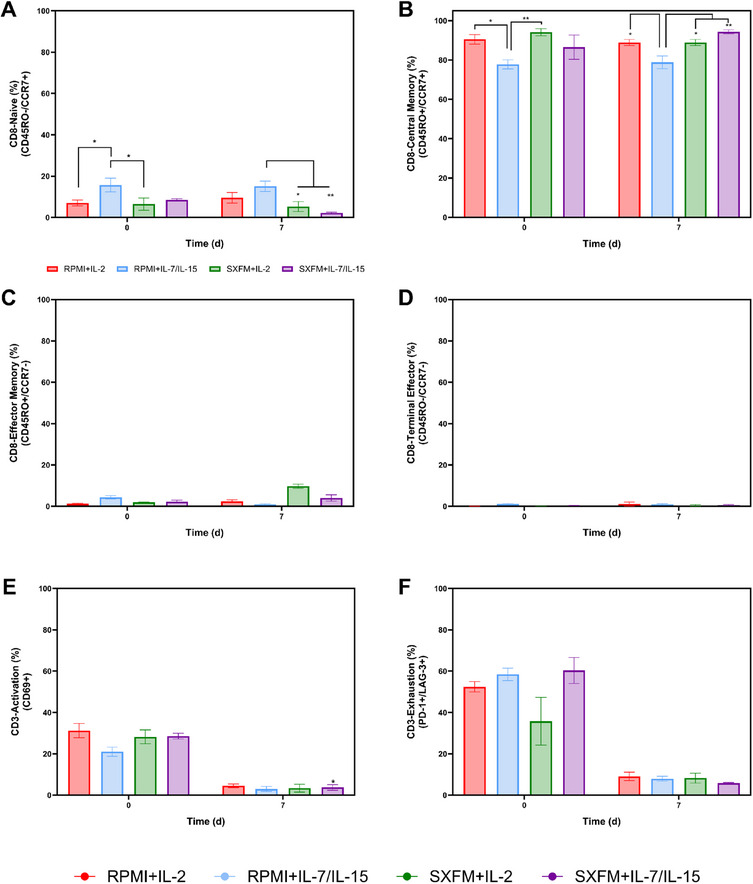
Representation of the different CD8 subsets at seeding and harvesting: (A) Naïve, (B) Central Memory, (C) Effector Memory and (D) Terminal Differentiated as well as (E) activation and (F) exhaustion profile of the CD3 populations for the static study. Vertical bars indicate the mean, with error bars representing one standard deviation (*N* = 3).

Regarding the CD8 subsets generated within the experimental design, RPMI supplemented with IL‐7/IL‐15 resulted in a higher fraction of naïve T cells (CD8⁺/CD45RO^‐^/CCR7⁺) (Figure 5A). However, this difference was evident from the beginning of the expansion phase, suggesting it was present before the expansion. Conversely, this same process condition led to a lower central memory fraction (CD8⁺/CD45RO^‐^/CCR7⁺), also identified at the beginning of the expansion stage (Figure 5B). Effector memory (CD8⁺/CD45RO^+^/CCR7^−^) (Figure 5C) and terminally differentiated (CD8⁺/CD45RO^‐^/CCR7^−^) (Figure 5D) fractions did not appear to be affected by the experimental conditions in this static study. A common response was observed across all conditions, with a decrease in activation marker expression (CD3+CD69+) from seeding to harvest. In parallel, expression of PD‐1 and LAG‐3 also decreased over time, suggesting these markers were transiently upregulated due to activation rather than indicative of true exhaustion (Figure 5E,F).

### 3.2 CAR‐T Expansion in Agitated Conditions

**FIGURE 6:**
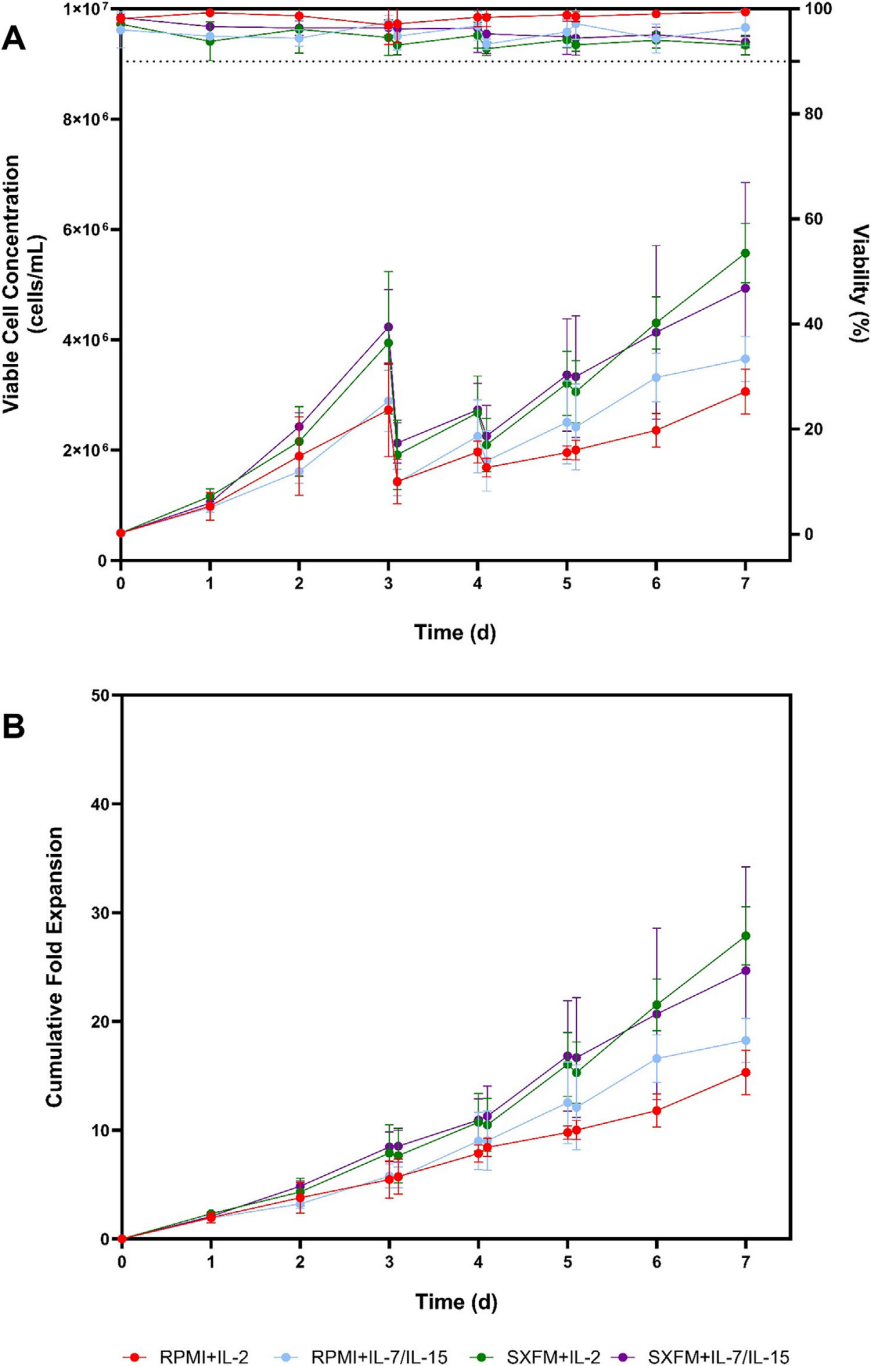
(A) Viable cell concentration and viability and (B) cumulative fold expansion for STR‐based expansion processes. Data points indicate the mean and errors bars represent one standard deviation (*N* = 3).

Given the G‐Rex platforms' limited monitoring and control capabilities, a 500 mL stirred tank platform was used to evaluate the impact of medium formulation and cytokine combinations on the CAR‐T manufacturing process. Several differences were noticed across multiple batches, reflecting the impact of medium, cytokine combination and donor variability on the process (Figure 6A,B). Irrespective of the process condition, the viability levels remained above 90% throughout the expansion phase. In addition, it was consistently observed that cultures supplemented with SXFM achieved higher cell concentrations compared to those supplemented with RPMI, highlighting the enhanced growth‐supporting properties of SXFM under agitated culture. In this study, the maximum cell yields observed ranged between 3.06 × 10^6^ and 5.56 × 10^6^ cells.mL^−1^. This was also evident in the differences in doubling time and growth rate (Table S1).

**FIGURE 7:**
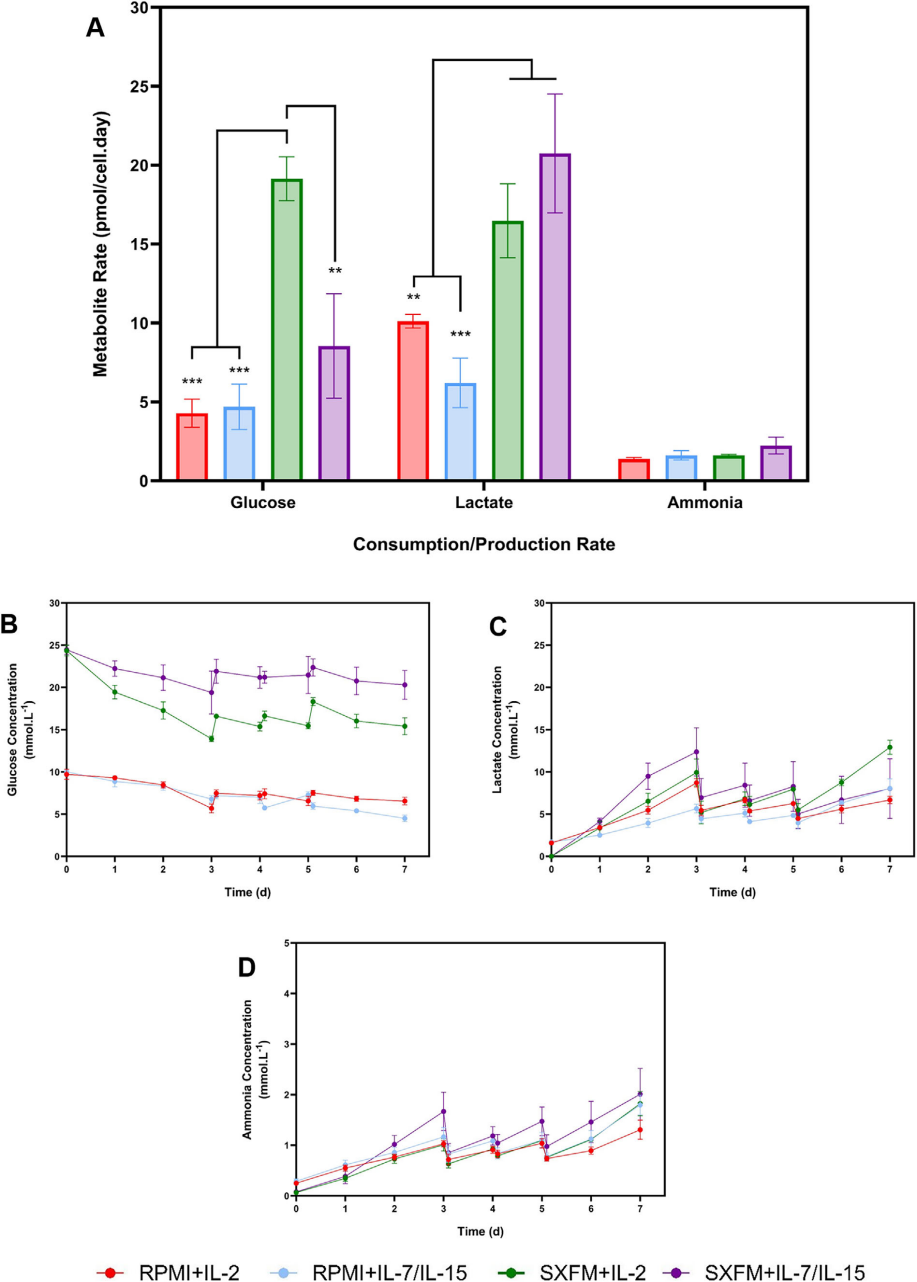
(A) Representation of the metabolite consumption and production rate and the concentration profiles for (B) glucose, (C) lactate and (D) ammonia in mmol.L^‒1^ for the STR‐based process. Vertical bars and points indicate the mean, with error bars representing one standard deviation (*N* = 3).

Several differences were identified in the metabolic consumption/production rate, particularly for glucose and lactate (Figure 7A). There was no clear indication of whether the medium formulation drove these differences, the cytokines or a combination of both. The analysis of metabolite profiles demonstrated that glucose decreased over time without being depleted at any point within the process (Figure 7B). Conversely, a lactate build‐up was observed due to glucose metabolism. Nonetheless, its concentration remained below 10 mmol.L^−1^ (Figure 7C). Ammonia accumulated to a maximum concentration of approximately 2 mmol·L^‒1^ (Figure 7D).

**FIGURE 8:**
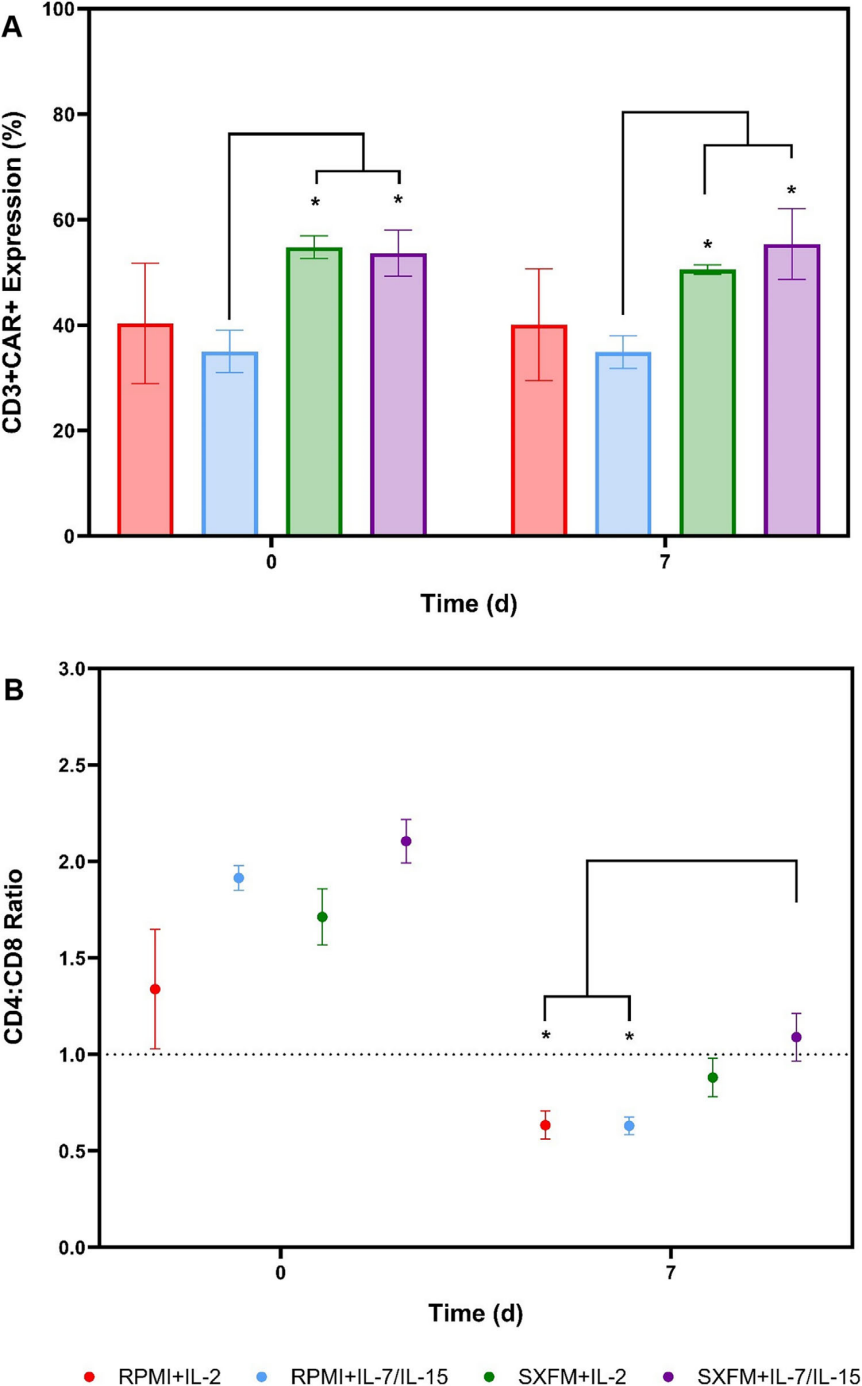
(A) CD3+/CAR+ expression and (B) CD4:CD8 ratio in the T‐cell populations at seeding and harvesting for the STR‐based process. Vertical bars and points indicate the mean with error bars representing one standard deviation (*N* = 3).

Recognising the importance of cytokine supplementation and medium formulation in T cell activation and their influence on transduction efficiency, this study focused on evaluating these factors. Transduction efficiencies obtained before the bioreactor‐based process ranged between 30% and 60%, with SXFM reporting higher transduction levels than its RPMI counterpart (Figure 8A). This indicates the benefit of using SXFM to enhance transduction levels in spinoculation‐based processes. As expected, given that a lentiviral vector was used in this work, CAR expression levels remained stable during the expansion phase, suggesting stable transgene integration. In addition, this finding suggests that CAR+ and non‐transduced cells are expanding at similar rates. To understand the impact of the bioprocess conditions on the final product quality, the immunophenotypic profile was assessed upon seeding (D0) and harvesting (D7). Similar to the observations in static cultures, cells manufactured using SXFM exhibited a more balanced CD4:CD8 ratio than those cultured in RPMI (Figure 8B).

**FIGURE 9:**
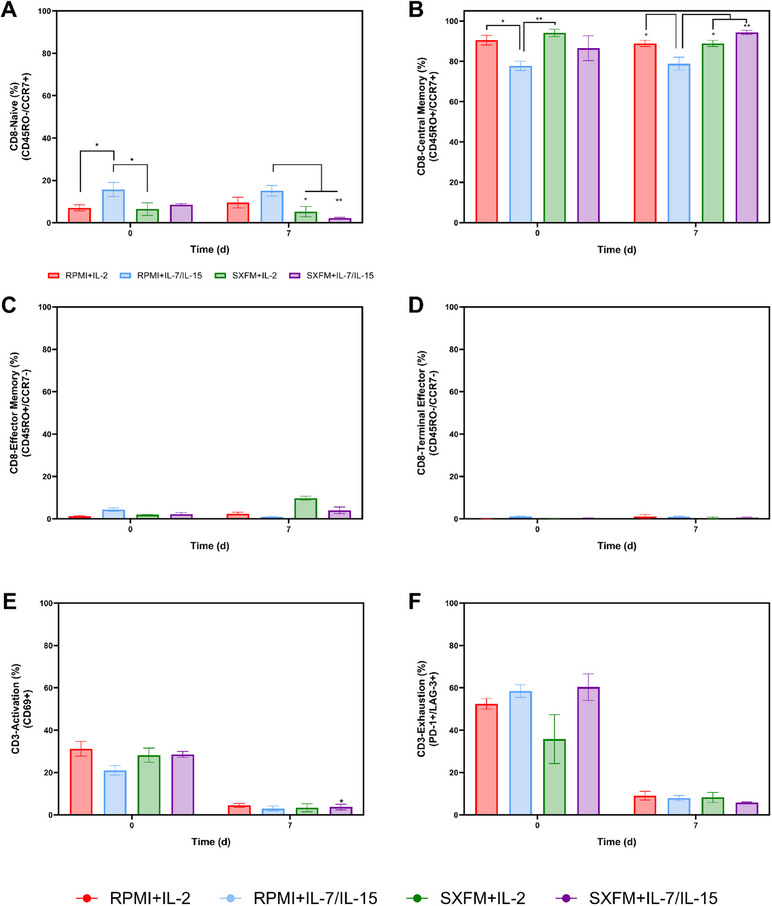
Representation of the different CD8 subsets at seeding and harvesting: (A) Naïve, (B) Central Memory, (C) Effector Memory and (D) Terminal Differentiated as well as (E) activation and (F) exhaustion profile of the CD3 populations for the STR‐based process. Vertical bars indicate the mean, with error bars representing one standard deviation (*N* = 3).

Irrespective of the medium and cytokine combination used, the stirred tank platform yielded approximately 90% of the CD8 cells either in their naïve (CD8⁺/CD45RO^‐^/CCR7⁺) or central memory CD8⁺/CD45RO^‐^/CCR7⁺ stage (Figure 9A,B, respectively). Conversely, the fraction of cells in the effector memory (CD8⁺/CD45RO^+^/CCR7^−^) and terminal effector (CD8⁺/CD45RO^‐^/CCR7^−^) remained below 10% at harvesting (Figure 9C,D, respectively). Helper T‐cells, CD4+ subsets observed a decrease in the naïve and central memory fractions during the expansion period at the expense of an increase in the effector memory (Figure S1).

A decrease in both the activation (CD3⁺/CD69⁺) and exhaustion (CD3⁺/PD‐1⁺/LAG‐3⁺) profiles was observed throughout the expansion phase (Figure 9E,F), which is expected due to the time elapsed since initial activation and its corresponding effect on these markers.

**FIGURE 10:**
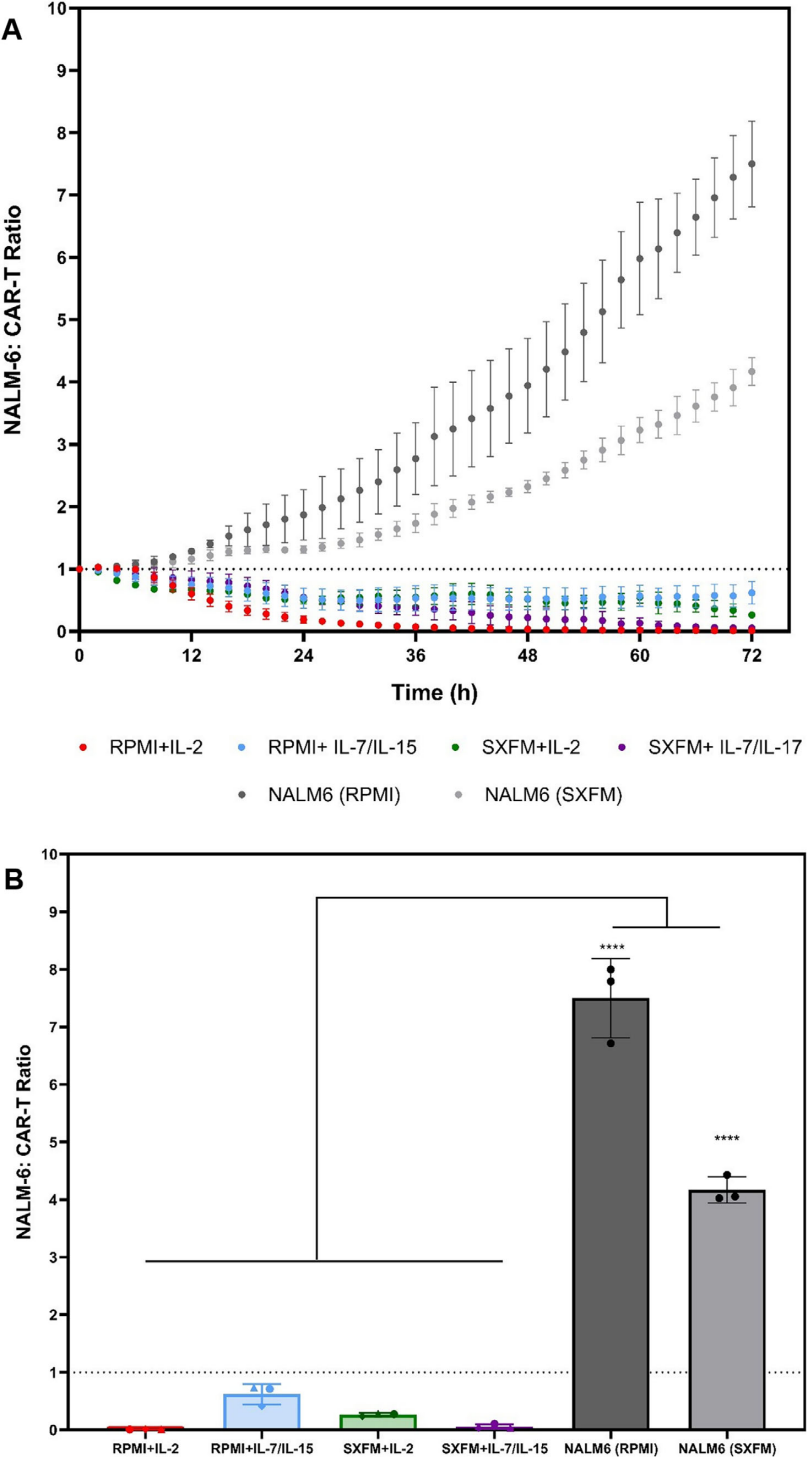
Evaluation of the in vitro cytotoxicity of the CAR+ populations generated across the different experimental conditions using a (A) time‐lapse analysis and a (B) comparison at the 72‐h mark from the STR‐based process. Points and vertical bars indicate the mean, with bars representing one standard deviation (*N* = 3). The dotted line represents the threshold whereby CAR‐T cell potency is observed.

In order to validate the in vitro potency of the generated CAR‐T cells regardless of the medium and cytokine combination used, a cytotoxicity assay was performed (Figure 10A,B). As expected, the negative controls resulted in exponential growth of the target cells (NALM‐6). In contrast, the co‐incubation with CAR‐T cells led to a decrease in tumour cells, indicating the product's cytotoxicity toward these. Target cell cytotoxicity was observed as a result of CD19 expression, which is specifically recognised by the CAR construct employed in this study. This effect was observed across all culture conditions tested, highlighting the versatility of the STR platform in consistently generating functional CAR‐T cells.

## 4 Discussion

This work demonstrated the feasibility of using SXFM under both static and agitated culture conditions, highlighting its potential benefits in improving growth kinetics, CD4:CD8 ratios and transduction efficiency. The static work conducted in the G‐Rex platforms demonstrated that the growth kinetics were statistically similar, irrespective of the medium and cytokine combination used. Using this expansion platform, the CAR‐T cells generated reached an expansion between 35 and 41 fold, aligned with previous research conducted using a similar setup [47, 48, 53, 54]. Interestingly, previous work using K562 cells has suggested that in the absence of nutrient depletion, Grex 24 well plates platform can accommodate a maximum of 14 × 10^6^ cells.cm^−2^ due to restrictions related to surface area. During the process, cell densities reached up to 18 × 10⁶ cells.cm^−^^2^, higher than what has been documented in previous studies. This can potentially be attributed to the differences in diameter across these two cell types [55], potentially explaining the absence of a concentration plateau in this work. From a metabolic perspective, glucose levels were nearly depleted by the end of the process in cultures supplemented with RPMI. In contrast, those using SXFM maintained levels above depletion, likely due to SXFM's substantially higher concentration of this carbon source in its formulation. Interestingly, none of the conditions tested led to a lactate concentration of 30 mM or above, the threshold previously described to affect cell viability negatively [54].

Regarding the immunophenotype at harvest, during the static G‐Rex studies, the SXFM maintained a one‐to‐one CD4:CD8 ratio, whereas FBS‐supplemented RPMI cultures exhibited a 1:2 ratio. However, it is worth noting that wider literature using FBS‐free or human serum‐containing formulations have reported even lower CD4:CD8 ratios. This suggests that additional factors, such as donor, activation, transduction, or expansion methodologies, may influence the final CD4:CD8 ratios achieved at the end of the process [48, 53, 54, 56, 57]. At harvest, the predominant phenotype obtained was central memory (∼80%), with naïve T‐cells representing 5%–10%. A similar differentiation breakdown was found in previous studies, both in G‐Rex and T‐flask, suggesting that factors other than the expansion platform play a role in the product quality at harvest [48].

In the absence of significant differences observed in static culture, which demonstrates comparability between products manufactured with FBS‐supplemented and SXFM, the second part of this study focused on evaluating the impact of SXFM and cytokine supplementation in STRs. This investigation supports wider efforts toward developing a scalable manufacturing process on this type of expansion platform, with the potential to align with GMP guidelines [58, 59, 60].

In agitated conditions, it was demonstrated that the SXFM used herein led to significantly faster growth kinetics than the serum‐supplemented research‐grade medium. This was particularly evident when this medium was combined with IL‐2. The maximum growth rate for FBS‐based cultures was 0.422 ± 0.015 d^−1^ versus 0.448 ± 0.042 d^−1^ for IL‐7/IL‐15 supplemented cultures of RPMI and SXFM, respectively. It is worth highlighting that growth rates observed in this study align with previous experimental efforts involving fed‐batch cultures under both conditions [37, 38, 45, 61]. In addition, the maximum viable cell concentrations reported in this work (5 × 10^6^ to 6 × 10^6^ cells.mL^−1^) are aligned with the maximum reported in platforms such as culture bags, Xuri and Prodigy when operated with human serum‐based medium formulations [53]. Notably, the STR‐based process established in this study consistently yielded at least one dose of CAR‐T cells across all experimental conditions (Figure S2). Introducing a SXFM significantly increased manufacturing capacity, producing four to five doses, depending on donor characteristics and the cytokines used. Perfusion processes are widely employed in cell and gene therapy manufacturing to enhance productivity by supporting higher cell concentrations and enabling continuous product harvesting [62, 63]. Recent studies have shown that incorporating perfusion culture into CAR‐T manufacturing achieves cell concentrations of approximately 20 × 10^6^ cells.mL^−1^ [45]. This advancement in manufacturing will be particularly crucial as allogeneic CAR‐T products progress to late‐stage clinical trials.

Regarding the final production quality, similarly to the observations for static culture, SXFM consistently maintained a CD4:CD8 ratio closer to one, aligning with the desired characteristics of CAR‐T products to maximise clinical efficacy [64, 65, 66]. On the other hand, RPMI‐supplemented medium led to a ratio closer to 1:2, which is aligned with previous reports found in the literature [37, 38].

The differentiation stage of CD8 subpopulations remained unaffected by the choice of medium and cytokine supplementation. Notably, most cells exhibited phenotypes ranging from naïve to central memory, mirroring the patterns observed in static culture. Interestingly, broader literature has demonstrated that expansion platforms may impact the characteristics of the harvested product, namely, in regards to the differentiated state of the CD4/CD8 subsets and activation, but not necessarily exhaustion [53]. Finally, using a SXFM did not change the immunophenotype of the cell product. It led to a similar exhaustion and activation profile compared to a research‐grade, FBS‐supplemented medium formulation. Finally, irrespective of the culture conditions, the CAR‐T products manufactured during this work exhibited in vitro cytotoxicity towards NALM6, a CD19‐based target cell type. Future studies will incorporate cytokine secretion analyses to provide a more sensitive and granular assessment of functional differences associated with manufacturing variations. Although this number is typical for early‐stage optimisation studies, further validation with a larger donor pool would confirm the broader applicability of the findings and accurately capture potential inter‐donor variability. In addition, T cell isolation in this study was performed using a research‐grade negative selection kit, which represents a limitation of the current workflow. Future iterations of this process will adopt GMP‐aligned positive selection methods (e.g., CD4⁺ and CD8⁺ isolation) to better align with clinical manufacturing standards. The establishment of an end‐to‐end, GMP‐compliant manufacturing process for CAR‐T cell therapies has the potential to significantly reduce production costs for both autologous and allogeneic applications. To achieve this goal, several stages need to be completed: (1) establishing a SXF process using reagents compliant with GMP standards (this work), (2) developing a fully closed end‐to‐end process, and (3) ensuring modularity by integrating gene delivery and fill‐and‐finish processes with the expansion platform. The inclusion of RPMI/FBS as a comparator was to establish a research‐grade reference point to benchmark the performance of the SXFM. The intention was not to suggest that RPMI/FBS represents a GMP‐compliant approach, but rather to assess how SXFM performs relative to a widely used laboratory standard. The replacement of FBS with a serum‐free medium represents an important step toward regulatory alignment and clinical translation.

Operating in closed systems directly impacts the required cleanroom environment and, therefore, the process's overall costs. FDA guidelines suggest that open systems, often involving manual interventions in open processes during the process, require higher‐grade cleanroom classifications (e.g., Grade B or ISO 7) to mitigate contamination risks [67]. Due to the more stringent requirements for maintaining sterility, semi‐closed and open processes are associated with increased operational costs.

Although some newly developed expansion platforms have integrated monitoring capabilities, they still lack essential control capabilities for parameters such as pH and DO. These factors are critical for regulating the manufacturing process, as fluctuations can significantly impact the final product's quality [51, 68, 69]. These findings demonstrate that SXFM supplemented with IL‐2 or IL‐7/IL‐15 supports the generation of high‐quality CAR‐T cell products in both G‐Rex and STR platforms, while addressing key manufacturing challenges associated with FBS use, including supply chain limitations, process variability, and pathogen transmission risks.

## 5 Conclusion

This study demonstrates that SXFM, particularly when supplemented with IL‐2, accelerated proliferation, enhanced transduction efficiency, and a higher CD4:CD8 ratio than serum‐containing formulations. From a manufacturing standpoint, this medium formulation maximises safety given the absence of any animal components. The final CAR‐T cells also maintained the desired immunophenotypic profiles and demonstrated in vivo cytotoxicity against CD19‐positive target cells. This work further demonstrates that SXFM supplemented with IL‐2 or IL‐7/IL‐15 enables scalable, reproducible, and cost‐effective CAR‐T cell manufacturing in STRs. Additionally, operating this process as a closed system may reduce the requirement for high‐grade cleanroom environments. Overall, these findings highlight the feasibility of using SXFM‐based processes to support GMP‐aligned CAR‐T cell manufacturing, providing a foundation for broader clinical translation and scalable production strategies for both autologous and allogeneic therapies.

## Author Contributions

Pedro Silva Couto conceptualised and designed the study with support from Dale Stibbs and Qasim Rafiq. All authors contributed to designing the study's methodology. Pedro Silva Couto and Dale Stibbs conducted the research and data collection. Pedro Silva Couto and Dale Stibbs were primarily responsible for data analysis and interpretation, with Pierre Springuel's contributions. Pedro Silva Couto and Dale Stibbs wrote the manuscript. Ursula Schultz contributes towards editing and improving the manuscript. All authors reviewed and edited the final draft. Qasim Rafiq supervised the project and gathered funding for the experimental execution. All authors have read and agreed to the published version of the manuscript.

## Conflicts of Interest

At the time of submission, Pedro Silva Couto, Dale J. Stibbs, Pierre Springuel, Stephen Goldrick, and Qasim A. Rafiq were affiliated with UCL. Sergio Navarro‐Velázquez and Manel Juan were with Hospital Clínic Barcelona, while Laura Herbst and Bastian Nießing were with the Fraunhofer Institute for Production Technology IPT. Katrin Mestermann, Carmen Sanges, and Michael Hudecek were affiliated with the Fraunhofer Institute for Cell Therapy and Immunology, and Ursula Schultz and Manuel Effenberger were employees of Sartorius CellGenix GmbH.

## Supporting information




**Supporting File 1**: biot70114‐sup‐0001‐SuppMat.docx.

## Data Availability

All data generated or analysed during this study is included in this published article.
