# Supplementary material for: Impact of Serum/Xeno‐Free Medium and Cytokine Supplementation on CAR‐T Cell Therapy Manufacturing in Stirred Tank Bioreactors
Source: Biotechnol J. 2025 Sep 9;20(9):e70114. doi: 10.1002/biot.70114 (PMC12419138; doi:10.1002/biot.70114)
Supplement: Supplementary file 1 — Supporting File 1: biot70114‐sup‐0001‐SuppMat.docx. [file BIOT-20-e70114-s001.docx]

**Supplementary table 1**

| **Culture Condition** | **Process Parameter** | **G-Rex 24 well plate** | **STR (500 mL)** |
| --- | --- | --- | --- |
| **RPMI+IL-2** | Maximum cell concentration (×10^6^.mL^-1^) | 6.62±0.44 | 3.06±0.41 |
|  | Total cell yield (× 10^6^ cells) | 52.90±3.11 | 766±101 |
|  | Doubling time (d) | 34.72±0.62 | 45.19±1.67 |
|  | Specific growth rate (d^−1^) | 0.479±0.008 | 0.368±0.013 |
|  | Cumulative Fold expansion | 46.31±3.10 | 15.32±2.02 |
|  | Glucose consumption rate (pmol.cell^−^1.d^−1^) | 112.71±32.59 | 4.28±0.894 |
|  | Lactate production rate (pmol.cell^−1^.d^−1^) | 217.62±20.41 | 10.11±0.43 |
|  | L-glutamine consumption rate (pmol.cell^−1^·d^−1^) | 108.38±2.72 | 1.43±0.53 |
|  | Ammonia production rate (pmol.cell^−1^.d^−1^) | 38.26±2.41 | 1.38±0.11 |
|  | CD4:CD8 ratio | 0.57±0.11 | 0.63±0.07 |
|  | CD3+/CAR+ (%) | 33.18±3.0 | 40.1±10.58 |
|  | % Naïve CD8 (CCR7+/CD45RO-) | 9.54±2.54 | 8.4±1.65 |
|  | % Central Memory CD8 (CCR7+/CD45RO+) | 88.87±1.53 | 73.53±3.94 |
| **RPMI+IL-7/15** | Maximum cell concentration (×10^6^.mL^-1^) | 6.37±0.16 | 3.65±0.41 |
|  | Total cell yield (× 10^6^ cells) | 51.00±1.02 | 913±102 |
|  | Doubling time (d) | 35.04±0.21 | 39.41±1.41 |
|  | Specific growth rate (d^−1^) | 0.474±0.002 | 0.422±0.015 |
|  | Cumulative Fold expansion | 41.53±0.64 | 18.26±2.04 |
|  | Glucose consumption rate (pmol.cell^−^1.d^−1^) | 117.43±35.30 | 4.69±1.44 |
|  | Lactate production rate (pmol.cell^−1^.d^−1^) | 289.09±8.90 | 6.20±1.57 |
|  | L-glutamine consumption rate (pmol.cell^−1^·d^−1^) | 37.16±0.61 | 1.01±0.69 |
|  | Ammonia production rate (pmol.cell^−1^.d^−1^) | 40.70±1.05 | 1.62±0.29 |
|  | CD4:CD8 ratio | 0.46±0.07 | 0.63±0.04 |
|  | CD3+/CAR+ (%) | 15.10±1.00 | 34.9±3.12 |
|  | Naïve CD8 (CCR7+/CD45RO-) (%) | 15.10±2.52 | 5.17±2.48 |
|  | Central Memory CD8 (CCR7+/CD45RO+) (%) | 78.80±3.25 | 93.10±1.74 |
| **TCM+IL-2** | Maximum cell concentration (×10^6^.mL^-1^) | 5.93±0.91 | 5.57±0.54 |
|  | Total cell yield (× 10^6^ cells) | 47.50±0.64 | 1390±135 |
|  | Doubling time (d) | 35.71±0.15 | 34.08±1.10 |
|  | Specific growth rate (d^−1^) | 0.466±0.002 | 0.488±0.016 |
|  | Cumulative Fold expansion | 41.54±0.64 | 27.88±2.70 |
|  | Glucose consumption rate (pmol.cell^−^1.d^−1^) | 188.98±39.73 | 19.15±1.4 |
|  | Lactate production rate (pmol.cell^−1^.d^−1^) | 249.65±4.51 | 16.45±2.35 |
|  | L-glutamine consumption rate (pmol.cell^−1^·d^−1^) | 29.05±2.48 | 6.74±0.16 |
|  | Ammonia production rate (pmol.cell^−1^.d^−1^) | 16.70±2.84 | 1.62±0.05 |
|  | CD4:CD8 ratio | 1.20±0.06 | 0.88±0.11 |
|  | CD3+/CAR+ (%) | 46.3±1.66 | 50.53±0.91 |
|  | Naïve CD8 (CCR7+/CD45RO-) (%) | 5.29±2.41 | 7.52±1.75 |
|  | Central Memory CD8 (CCR7+/CD45RO+) (%) | 88.88±1.53 | 89.93±0.86 |
| **TCM+IL-7/15** | Maximum cell concentration (×10^6^.mL^-1^) | 5.83±0.15 | 4.94±1.96 |
|  | Total cell yield (× 10^6^ cells) | 46.6±0.79 | 1230±478 |
|  | Doubling time (d) | 35.88±0.19 | 37.3±3.55 |
|  | Specific growth rate (d^−1^) | 0.464±0.003 | 0.448±0.042 |
|  | Cumulative Fold expansion | 40.81±0.79 | 24.68±9.56 |
|  | Glucose consumption rate (pmol.cell^−^1.d^−1^) | 183.04±25.26 | 8.55±3.32 |
|  | Lactate production rate (pmol.cell^−1^.d^−1^) | 331.08±45.04 | 20.74±3.77 |
|  | L-glutamine consumption rate (pmol.cell^−1^·d^−1^) | 322.29±14.68 | 3.77±0.96 |
|  | Ammonia production rate (pmol.cell^−1^.d^−1^) | 38.72±1.16 | 2.24±0.54 |
|  | CD4:CD8 ratio | 1.09±0.11 | 1.09±0.12 |
|  | CD3+/CAR+ (%) | 17.13±2.68 | 55.34±6.72 |
|  | Naïve CD8 (CCR7+/CD45RO-) (%) | 2.20±0.36 | 10.2±1.05 |
|  | Central Memory CD8 (CCR7+/CD45RO+) (%) | 94.33±1.00 | 81.43±0.58 |

**Supplementary Figure 1**

**
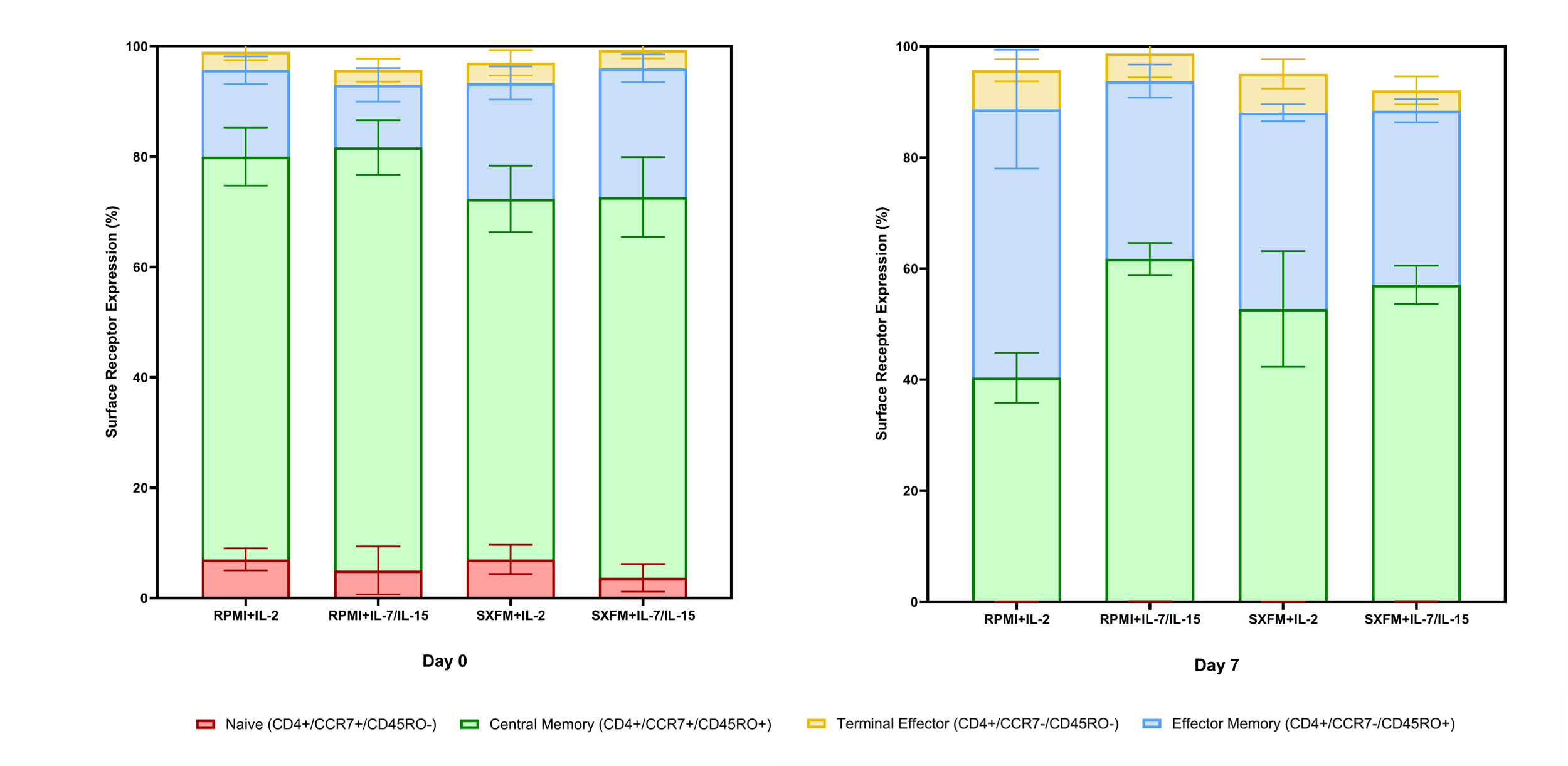
**

**Supplementary Figure 2**

**
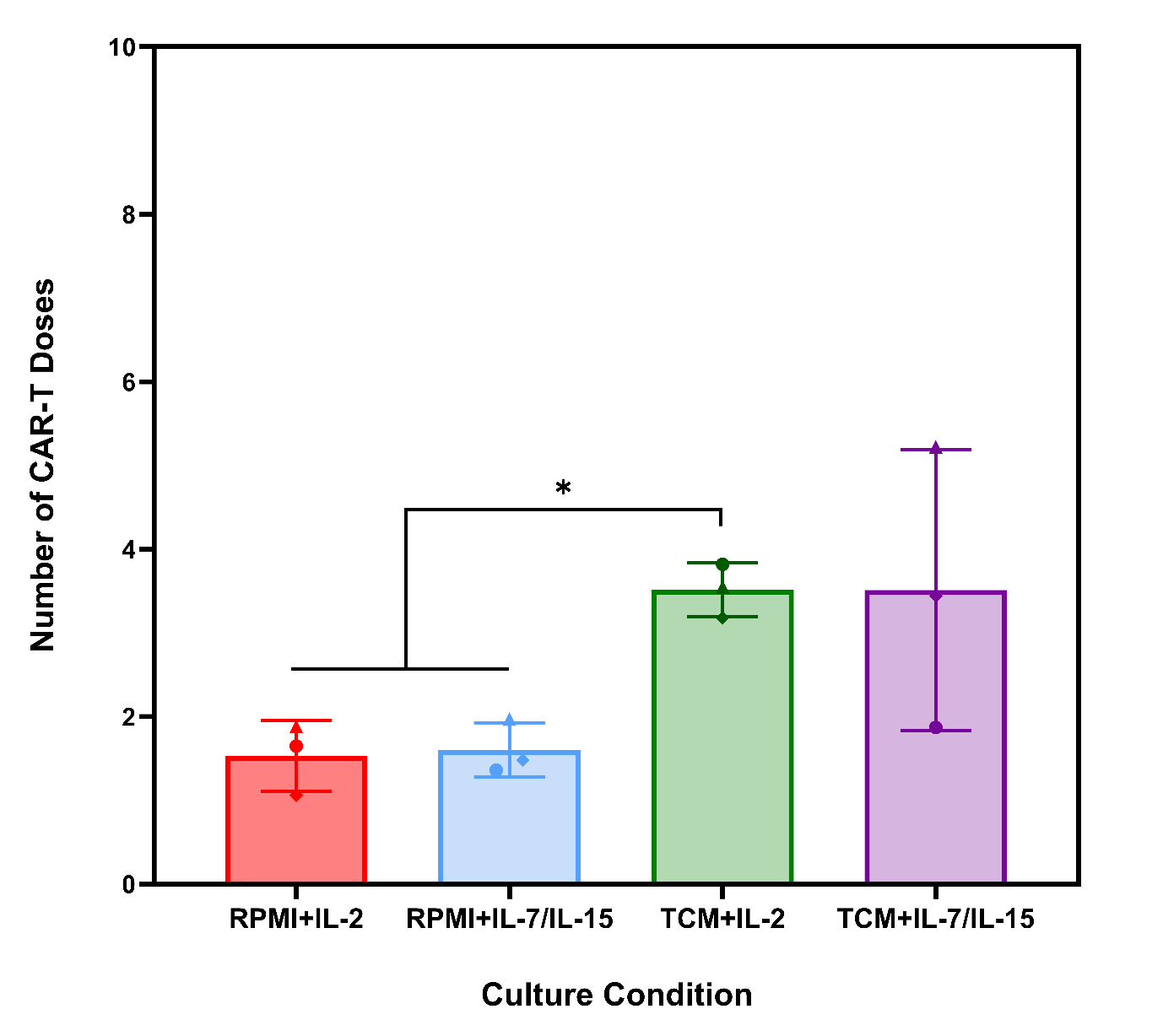
**
